# Population implications of the deployment of novel universal vaccines against epidemic and pandemic influenza

**DOI:** 10.1098/rsif.2019.0879

**Published:** 2020-03-04

**Authors:** N. Arinaminpathy, S. Riley, W. S. Barclay, C. Saad-Roy, B. Grenfell

**Affiliations:** 1 MRC Centre for Global Infectious Disease Analysis, Faculty of Medicine, Imperial College London, London, UK; 2 Department of Infectious Disease, Faculty of Medicine, Imperial College London, London, UK; 3 Lewis-Sigler Institute for Integrative Genomics, Princeton University, Princeton, NJ, USA; 4 Department of Ecology and Evolutionary Biology, Princeton University, Princeton, NJ, USA

**Keywords:** influenza, epidemiology, mathematical modelling, vaccine

## Abstract

There is increasing interest in the development of new, ‘universal’ influenza vaccines (UIVs) that––unlike current vaccines––are effective against a broad range of seasonal influenza strains, as well as against novel pandemic viruses. While the existing literature discusses the potential epidemiological benefits of UIVs, it is also important to anticipate their potential unintended population consequences. Using mathematical modelling, we illustrate two such types of adverse consequences. First, by reducing the amount of infection-induced immunity in a population without fully replacing it, a seasonal UIV programme may permit larger pandemics than in the absence of vaccination. Second, the more successful a future UIV programme is in reducing transmission of seasonal influenza, the more vulnerable the population could become to the emergence of a vaccine escape variant. These risks could be mitigated by optimal deployment of any future UIV vaccine: namely, the use of a combined vaccine formulation (incorporating conventional as well as multiple universal antigenic targets) and achieving sufficient population coverage to compensate for any reductions in infection-induced immunity. In the absence of large-scale trials of UIVs, disease-dynamic models can provide helpful, early insights into their potential impact. In future, data from continuing vaccine development will be invaluable in developing robustly predictive modelling approaches.

## 1. Introduction

Current vaccines against influenza have had considerable impact [1], but their efficacy depends on a close match between the vaccine and the circulating influenza virus, in particular to the immunodominant ‘head’ region of the viral surface protein haemagglutinin (HA). Current vaccines therefore have to be updated regularly to remain effective in the face of viral evolution; they also cannot be used for pandemic mitigation, owing to the unpredictability of pandemic viruses and the long (six month) development time of current vaccines.

In recent years, there has been increasing interest in new vaccines against alternative antigenic targets that can provide more broad, long-lasting protection against seasonal and pandemic influenza viruses [2,3]. Several such ‘universal’ influenza vaccines (UIVs) are currently in development (table 1) [14]; they may offer qualitatively new opportunities for influenza control, raising the prospect of routine vaccination programmes that do not need to be updated as often, while also protecting against novel pandemic viruses.

**Table 1.** Summary of different immune targets for influenza vaccines. Among current influenza vaccines, ‘inactivated’ vaccines focus on HA1 immunity (top row), while ‘live attenuated’ vaccines could raise both HA-specific and T-cell immunity. However, their heterosubtypic protection is unclear. The last two rows correspond to strategies being pursued for the development of new, ‘universal’ vaccines (we adopt the scenario in the bottom row for the purpose of the current work).

antigenic target	type of immunity	breadth of immunity	Relevant sources
haemagglutinin (HA1, head region)	sterilizing (reducing susceptibility)	strain-specific and immunodominant; seasonal vaccines need to be updated regularly and are not effective against novel pandemic strains	[4,5]
haemagglutinin (HA1, conserved epitopes in head region)	sterilizing (reducing susceptibility)	vaccine targets identified through computational methods and may offer broad, within-subtype protection	[6,7]
haemagglutinin (HA2, stalk region)	sterilizing (reducing susceptibility)	broad protection within and across subtypes (animal models): could offer pandemic protection in humans	[8–10]
T-cell antigens, e.g. matrix proteins (M1 and M2), nucleoprotein (NP)	non-sterilizing, but could reduce clinical severity, and potentially infectiousness, by limiting viral load	broad protection within and across subtypes (animal models): could offer pandemic protection in humans	[11–13]

Previous evidence from disease-dynamic models illustrated the possible benefits of mass deployment of UIVs [15,16]. However, population immunity to influenza is complex and incompletely understood, raising the possibility of unintended consequences from UIVs. It is important to anticipate these effects and determine how they might be mitigated. Here, using a simple mathematical model capturing some essential features of influenza transmission and immunity, we present two theoretical examples of how a UIV programme could exacerbate a population's vulnerability to influenza. In the face of uncertainties surrounding influenza immunity, we discuss key areas that future work should address in order to develop robust quantitative estimates for the impact of future vaccines.

## 2. Material and methods

We focus on the USA, which currently has the world's largest population (all-age) coverage of seasonal influenza vaccination [17]. Building on earlier work [18], we develop a simple model of influenza immunity and transmission dynamics in the USA (see electronic supplementary information for technical details). The model incorporates two types of immunity, as follows. (i) HA-specific immunity, which reduces susceptibility to infection and is acquired either through past infection or through effective strain-matched vaccination. This immunity is strain-specific and does not protect against novel pandemic viruses. (ii) Cross-protective immunity, which is assumed to be independent of HA-specific immunity and is likewise acquired either through past infection or through a UIV. This immunity is heterosubtypic: that is, it offers protection across different subtypes. Moreover, we assume that cross-protective immunity does not affect susceptibility to infection, but rather limits viral load during the course of infection, thus reducing infectiousness. This would be consistent, for example, with a UIV targeting T-cell antigens [19]; however, we note that HA-stem antibodies would be expected to offer some protection against infection [20,21] (table 1).

We simulate a population that is subject to seasonal UIV vaccination, and subsequently exposed to a 2012/13-like season (a severe season in the USA). Having prepared a ‘test population’ in this way through combined immunization events (the routine UIV programme and the seasonal epidemic), we then assess the vulnerability of this test population to different types of subsequent immune escape.

## 3. Results

**Figure 1.**
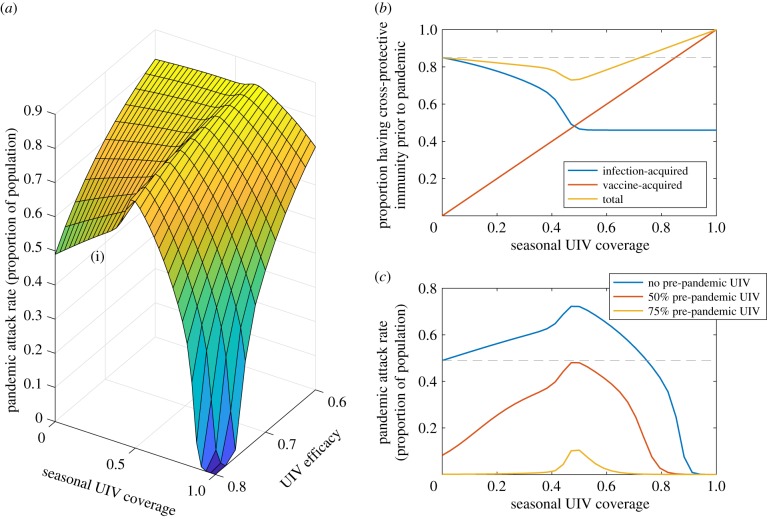
Implications of routine seasonal UIV for a population's vulnerability to pandemic influenza. Here and throughout, we define the ‘efficacy’ of a UIV as the percentage drop in transmission potential arising from vaccination. (*a*) Pandemic size under a range of values for seasonal UIV coverage and efficacy (assuming no vaccination response against the pandemic). The rising edge marked (i) illustrates that low seasonal UIV coverage, especially for an efficacious vaccine, can inadvertently increase pandemic attack rates. (*b*) How cross-protective immunity in the test population is affected by seasonal UIV coverage, at 80% UIV efficacy (i.e. the edge marked (i) in (*a*)). Vaccination brings down the amount of infection-acquired immunity in the test population (blue curve). At low coverage, the vaccination programme fails to compensate for this loss of immunity (yellow curve, initial decline). The dashed grey line indicates the level of cross-protective immunity in the absence of the UIV programme; this is only exceeded by a UIV coverage of at least 75%. (*c*) Relaxing the assumption of no pre-pandemic vaccination, again taking the cross-section corresponding to a UIV efficacy of 80%. As in (*b*), the horizontal grey line indicates the pandemic size in the absence of vaccination.

We begin by examining population vulnerability to *pandemic emergence*. Figure 1 shows one illustrative scenario, where the test population is exposed to a pandemic virus (to begin, we assume there is no supplementary UIV programme mounted in direct response to the pandemic). At low levels of UIV coverage, a seasonal UIV programme may in fact increase the pandemic size. Figure 1*b* illustrates reasons for this behaviour. By bringing down seasonal incidence, the UIV programme reduces opportunities for individuals to acquire infection-induced immunity. As a result, there is an expanded pool of individuals who have been neither vaccinated nor infected, and who are thus vulnerable to the emergence of a pandemic virus. However, the figure also illustrates that it is possible to overcome this population effect if a seasonal UIV programme has sufficient coverage. In particular, a UIV with 80% efficacy and 75% coverage would not only interrupt transmission for the 2012/13-like season, but would also result in a mitigated pandemic, relative to the absence of vaccination. Electronic supplementary material, figure S2 shows results disaggregated by age, illustrating similar dynamics in each of the age groups.

In figure 1*c*, we relax the assumption of no pre-pandemic vaccination. Overall, if seasonal UIV programmes can increase a population's vulnerability to new pandemic viruses, figure 1*b*,*c* illustrates two ways in which it is possible to counteract this effect: with sufficiently high vaccination coverage either in seasonal UIV programmes (figure 1*b*), or in pre-pandemic UIV programmes (figure 1*c*).

**Figure 2.**
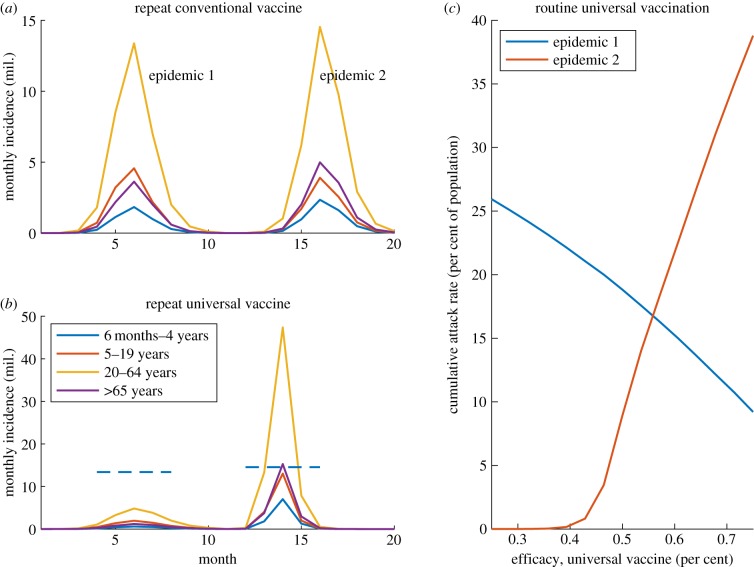
Potential impact of UIV vaccine failure. Here, ‘epidemic 1’ denotes the 2012/13-like season used to prepare the test population, and we assume escape to cross-protective immunity occurs immediately after this epidemic. (*a*) With routine seasonal vaccination using a conventional (strain-matched) vaccine, epidemic sizes are unchanged by UIV immune escape. (*b*) With routine seasonal vaccination using a UIV, successful control of epidemic 1 can have the unintended effect of permitting a larger epidemic 2. For comparison, dashed lines show the epidemic peaks reached under a conventional vaccine (*a*). (*c*) How epidemic sizes in (*b*) change with UIV efficacy in epidemic 1 (we assume throughout that this efficacy declines to 25% in epidemic 2, as a result of vaccine escape).

We next examine population vulnerability to a *UIV escape variant*: that is, a virus showing vaccine escape to all UIV antigenic targets but remaining susceptible to existing HA-specific immunity. Figure 2 shows the scenario where the test population is exposed to such a UIV escape variant. The figure shows two scenarios: where routine vaccination is conducted using strain-matched vaccines (figure 2*a*) and where it is instead conducted using a UIV (figure 2*b*). Under the first scenario, the two epidemics are of comparable size. Under the second scenario, although the UIV succeeds in controlling the initial epidemic, it does so at the expense of strain-specific immunity. As a result the subsequent season, associated with a UIV escape mutant, is considerably larger than it otherwise would have been (the latter indicated by the dashed horizontal lines for comparison). As discussed below, such risks could be mitigated by a combined vaccination strategy.

## 4. Discussion

By focusing on specific types of immunity over others, a vaccination programme may shift the landscape of population immunity in unexpected ways, particularly in settings such as the USA, where routine coverage already exceeds 40% of the population [17]. The development of universal influenza vaccines is still in its early stages; in the absence of large-scale trials, mathematical modelling can be a helpful tool for generating some preliminary insights into the potential population implications of these vaccines. Here we have illustrated two potential risks.

First, a UIV could in principle control seasonal epidemics, without fully compensating (at the population level) for the reduced opportunities for individuals to gain infection-induced immunity (figure 1*b*). Doing so would leave the population more vulnerable to a pandemic. Indeed, a similar effect has been proposed among those receiving conventional vaccines [22]. Here we demonstrate ways in which these effects could even arise indirectly (i.e. through reducing transmission), thus adversely affecting even those who have not received the vaccine.

Second, by controlling seasonal epidemics, seasonal UIV programmes may also compromise strain-specific immunity in the population: this becomes a concern in the context of transmissible escape mutants to cross-protective immunity (figure 2). Despite strong arguments for why certain immune targets may face functional constraints preventing them from expressing significant variation [23,24], the possibility of escape cannot be fully discounted [25].

These risks could be mitigated to some extent by adequate planning of a future vaccination programme: for example, figure 1*a* suggests that a routine UIV programme could indeed mitigate against pandemics if it has a sufficiently high coverage to overcome the loss of infection-induced immunity. Figure 1*b* suggests a minimum coverage of 75%, a substantially higher threshold than the standard ‘critical vaccination threshold’ required to interrupt transmission of seasonal influenza (roughly 66% needing to be effectively vaccinated for a virus with *R*_0_ = 1.5) [26]. Likewise in the case of compromising strain-specific immunity, an approach that deploys conventional, strain-matched vaccines in parallel with UIVs, together with multiple UIV targets, could mitigate strongly against the risk of vaccine escape.

This model is a deliberately simplified and illustrative framework. We have necessarily ignored different potential immune complexities, for example the potential role of original antigenic seniority [27,28] and immune priming [29]. In §3 of the electronic supplementary information, we discuss these and other immune complexities that will form an important basis for future work to address.

In conclusion, although vaccination has offered great benefits in the control of influenza for several decades, new technologies may offer qualitatively new opportunities for influenza control. In view of the complex effects that UIVs could have on population immunity, the design of their ‘target product profiles' would benefit significantly from population dynamic perspectives.

## Supplementary Material

Additional technical information

## Data accessibility

This study used no data beyond that reported in the manuscript and in the electronic supplementary information.
