# Supplementary material for: Population implications of the deployment of novel universal vaccines against epidemic and pandemic influenza
Source: J R Soc Interface. 2020 Mar 4;17(164):20190879. doi: 10.1098/rsif.2019.0879 (PMC7115234; doi:10.1098/rsif.2019.0879)
Supplement: Additional technical information [file rsif20190879supp1.docx]

**Population implications of the deployment of novel universal vaccines against epidemic and pandemic influenza**

Arinaminpathy N^1^, Riley S^1^, Barclay W.S^2^, Saad-Roy C^3^, Grenfell B^4^

^1^ MRC Centre for Global Infectious Disease Analysis, Faculty of Medicine, Imperial College London, UK

^2^ Department of Infectious Disease, Faculty of Medicine, Imperial College London, UK

^3^ Lewis-Sigler Institute for Integrative Genomics, Princeton University, Princeton, USA

^4^ Department of Ecology and Evolutionary Biology, Princeton University, Princeton, USA

**Supporting Information**

1. ***Specification of the model***

We built on a model previously developed to capture the impact of seasonal influenza vaccination in the USA. The model framework is described in detail in ref [1]. In brief, the model is a deterministic, compartmental, age-structured framework, capturing the influenza epidemic in the USA at the national level, and allowing for different levels of prior immunity in each of the age groups.

*Modelling immunity*

As described in the main text, the model incorporates two types of immunity: (i) HA-specific immunity, which reduces susceptibility to infection, and (ii) Independently acting cross-protective immunity, assumed not to affect susceptibility to infection, but rather to reduce infectiousness (Table 1). For illustration we assumed vaccine-derived immunity to elicit identical protection as infection-induced immunity (in strength and duration), but focusing solely on specific antigens, whether HA-specific (as for conventional, strain-matched vaccines) or broadly protective (as for universal vaccines). For simplicity, we focus on inactivated vaccines and not live attenuated vaccines, the former of which account for the majority of influenza vaccination in the USA. Moreover we did not aim to model the dynamics of immunity across different influenza seasons, instead treating each epidemic independently. In the absence of serology or other immunological data, it is not possible to estimate separately the roles of HA-specific or cross-protective immunity, for a given epidemic; instead we assumed that a certain proportion of individuals have both cross-protective and strain-matched immunity in advance of the simulation, rendering them fully protected from infection. We modelled this effective prior immunity as acting in an all-or-nothing manner, thus governing the proportion of the population initially in the ‘susceptible’ vs ’recovered’ compartments (that is, through the initial conditions for the epidemic – see below for a discussion of the limitations of this approach). We estimated the age-specific proportions having this immunity using the data described above.

For simplicity we ignored dynamics such as ‘antigenic imprinting’, or the protection arising from childhood infection, against exposure later in life to heterosubtypic, zoonotic influenza viruses [3] (discussed further below). We also ignored the potential impact of universal vaccination on influenza evolution; this is consistent with genomic analysis suggesting that the principal source of antigenic novelty for influenza is not the USA, but in South-East Asia and in the tropics [4,5].

*Governing equations and calibration*

For a single season, we neglected births and deaths in the host population. In the following equations, we denote age groups with subscript *i,* where *i* = [1, 2, 3, 4] denote respectively 6 mo – 4 yrs, 5 – 19 yrs, 20 – 64 yrs, and >65 yrs. We denote vaccination status with the conventional (HA-based) vaccine as *j*, where *j* = [0, 1] denotes respectively unvaccinated and vaccinated individuals. Similarly we denote as $k$the vaccination status with the cross-protective vaccine. We write $S_{ijk},I_{ijk},R_{ijk}$for the proportions of the population that are, respectively, susceptible, infected and recovered, each stratified by the age and vaccination categories $i,j,k.$

Consistent with mechanisms of action discussed in the main text, we assume that strain-matched vaccination reduces susceptibility to infection by a proportion $p_{i}$ in age-group *i*, and cross-protective vaccination reduces infectiousness by a proportion $q_{j}$ in age-group *j*.

Governing equations for the model are:

$$\dot{S}_{ijk} = -\left( 1-jp_{i} \right)\beta\sum_{a,b,c} S_{ijk}m_{ia}\frac{I_{abc}}{N_{a}}\left( 1-{cq}_{a} \right) \dot{I}_{ijk} = \left( 1-{jp}_{i} \right)\beta\left[ \sum_{a,b,c} S_{ijk}m_{ia}\frac{I_{abc}}{N_{a}}\left( 1-{cq}_{a} \right) \right]-\gamma I_{ijk} \dot{R}_{ijk} = \gamma I_{ijk}$$

where $\gamma$ is the per-capita rate of recovery, and (for convenience of notation) the vaccine status indices *j*, *c* are being used as indicator functions (i.e. their values 0, 1 being treated not only as categorical, but also as multiplying terms in these equations).

Free model parameters, to be calibrated, included: the initial susceptibility in each age group (as a result of past, strain-specific exposure); the overall rate of transmission per day $\beta$; and the age-specific effect of vaccination in reducing susceptibility. Treating each influenza season independently, these parameters were estimated using the following data sources: virologically confirmed influenza hospitalisations in each month; age-specific estimates of vaccine efficacy (VE) for each season; and estimates of monthly, age-specific vaccine coverage in each season. Additionally, the model is informed by ‘multipliers’ estimated by the US Centers for Disease Control and Prevention (CDC), which relate hospitalisations to the incidence of symptomatic illness. In ref [1] we describe in detail how the model was fitted to this data using Bayesian methods. In the present work, which aims to illustrate qualitative dynamics, uncertainty estimates are less critical than in ref. [1]. For the simulations we therefore selected the best fitting parameter set for the 2012/13 season, i.e. that maximising the posterior density constructed as in ref [1]. Table S1 summarises these point estimates.

| Parameter |  | Values (age group $a$) | | | | Source |
| --- | --- | --- | --- | --- | --- | --- |
|  |  | 6mo – 4yrs | 5 – 19yrs | 20 – 64yrs | >65 yrs |  |
| Population size, $N_{a}$ | | 17879414 | 62505456 | 188263884 | 43145356 | U.S. Census Bureau, population division |
| Initial proportion having both cross-protective and strain-matched immunity, $R_{a}(0)/N_{a}$ (*) | | 0.17 | 0.54 | 0.47 | 0.20 | Model calibration to surveillance data [1] |
| Conventional vaccine efficacy, $p_{a}$ | | 0.55 | | 0.26 | |  |
| Per-capita rate of recovery, $\gamma$ | | 5 | | | | Assumption |
| Number $m_{ia}$ of daily contacts with age group $i$ | 6mo – 4yrs | 1.9 | 4.2 | 0.45 | 0.17 | Mossong et al [6], assuming same parameters as for UK |
|  | 5 – 19yrs | 1.8 | 8.8 | 1.9 | 1.2 |  |
|  | 20 – 64yrs | 5.0 | 5.5 | 7.9 | 4.6 |  |
|  | >65 yrs | 0.23 | 0.30 | 0.66 | 1.9 |  |

**Table S1. Summary of model parameters**, used to model the 2012/13 season in the USA as a ‘test epidemic’. Estimates are drawn from an earlier modelling study [1]. Whereas that study included uncertainty estimates, in the present work – which focuses more on illustration – we use only point estimates. Footnotes: (*) As described above, we model any population immunity prior to the simulation in a simple way, by assuming this immunity to act in an ‘all-or-nothing’ manner. Given these estimated values, initial conditions for susceptibility are calculated using: $S_{a}\left( 0 \right)=N_{a}-R_{a}\left( 0 \right).$

1. **Schematic of the approach**

The following figure illustrates schematically the modelling approach.


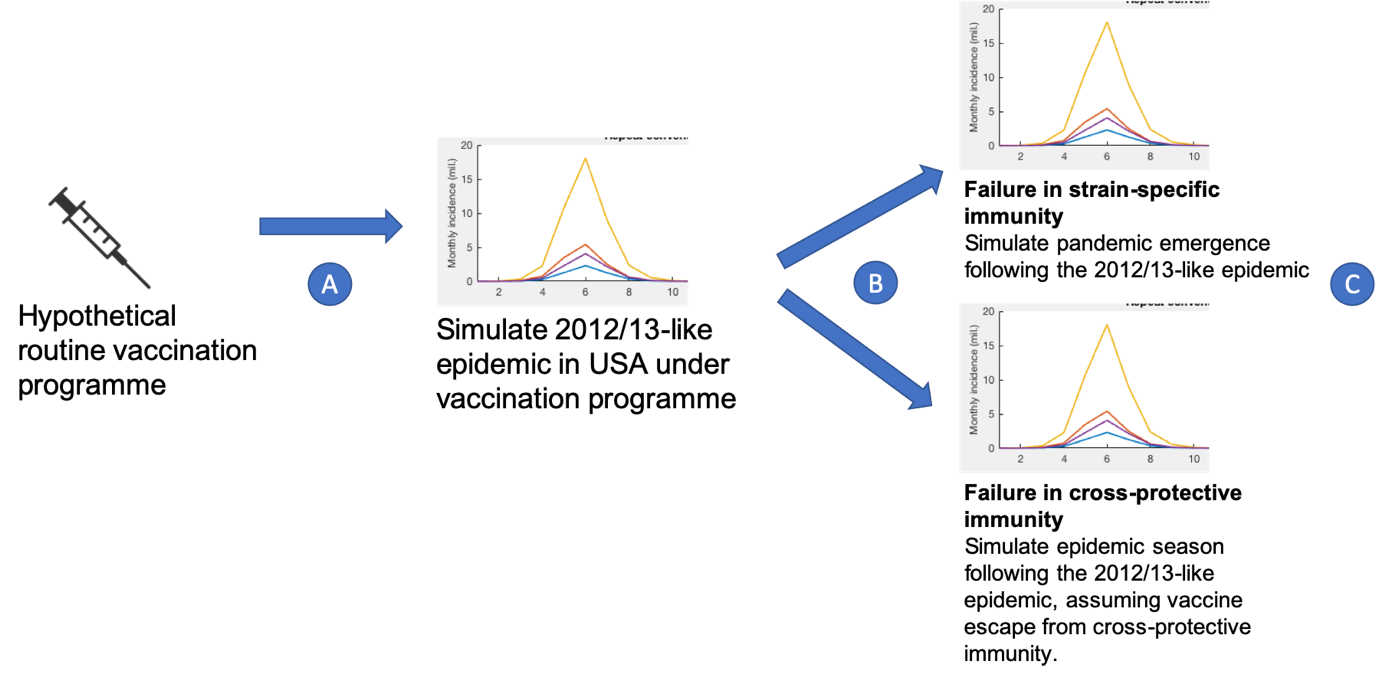


**Figure S1. Schematic of the modelling approach**. Points A, B, C mark stages where population immunity or epidemiological outcomes are assessed, as described below.

At stage (B) in the figure, population immunity depends on both the vaccination programme at stage (A), and the ensuing seasonal epidemic. However, the latter can be heavily influenced by the former; we use a dynamical transmission model to capture these relationships. In the main text, we refer to the population at stage B as a ‘test’ population. We then examine the behaviour of this test population under two types of exposure, illustrated on the right-hand side of the figure: (i) where a pandemic virus emerges soon after the epidemic season (upper right-hand plot), and (ii) alternatively, where a subsequent epidemic season is caused by a variant capable of escape from cross-protective immunity (lower right-hand plot). In each scenario, we assess how final epidemic sizes at stage (C) are shaped by the choice of vaccination programme at stage (A).

1. **Immune complexities and some key directions for future quantitative analysis**

In the model presented in the main text and described above, we adopted a deliberately simplified representation of influenza immunity. By capturing some essential interactions between infection and immunity, the model aims to demonstrate qualitative, dynamical behaviour. With improved understanding of influenza immunity and transmission, future modelling analysis should aim to incorporate more biological realism in order to derive more quantitatively predictive models of vaccine impact (for example, the ‘vaccination threshold’ illustrated in Figure 1B). Here we briefly survey some key factors in immunity against influenza, that would be important for future studies to address.

In section 3.1 we first list some limitations of the modelling approach, relating to our strategy for modelling immunity in the main text. Many of these factors hinge on the longitudinal dynamics of immunity against influenza: that is, how an individual’s immune response changes over their lifetime, including in response to vaccination. Given the importance of this topic, in section 3.2 below we present a brief survey of some of the relevant literature, and its potential role in future modelling of UIVs.

**3.1 Limitations of the modelling approach**

- We ignored any potential interactions between strain-specific and cross-protective immunity, for example the fact that B-cells can promote the cellular immune response, and vice versa [7,8]. With improved data on the implications of these mechanisms for transmission, future work could explore these interactions more closely, to examine their implications for vaccine development.
- For simplicity we assumed that prior exposure could be summarised through the proportion of the population having all-or-nothing immunity, in advance of the season. This approach, of combining both strain-matched and cross-protective immunity into a single status of ‘effective prior immunity’, was necessitated by the data available, which does not allow us to estimate separately the roles of strain-matched and cross-protective immunity. Future analysis will benefit from more systematic data on the typical distribution of cross-protective immunity in the population, including by age group.
- Our approach involves at most two successive epidemics: as such, it does not allow us to address the duration of cross-protective immunity elicited by immunisation, a property that will be an important characteristic of future UIVs [9]. There is a need for systematic studies to quantify this duration by monitoring cross-protective immunity over several years. In parallel, an important task for future modelling analysis will be to capture separately the dynamics of different arms of immunity over several seasons (see section 3.1 below).
- For simplicity we assumed vaccine- and infection-induced immunity to be identical. An important question for future work is the implication of vaccine-induced immunity that is inferior to infection-induced immunity: how should future vaccination programmes compensate for any such shortfalls? What aspects of immune protection (strength, breadth, duration, etc) are most important to address? To inform such analysis, cohort studies of individuals with different exposure and vaccination history would be invaluable.
- Other immune complexities that we have ignored include short-lived, ‘strain-transcending’ immunity that has been proposed to explain influenza evolutionary patterns [10]; the complex relationship between immunodominance and transmission-blocking [11]; and the potential impact of UIVs on seasonal influenza evolution [12].

**3.2 Long-term, longitudinal dynamics of immunity against influenza**

It has long been recognised that childhood exposure to influenza can have a strong influence on an individual’s immune response against influenza throughout life [13,14], a phenomenon originally termed ‘original antigenic sin’ [15]. Relatedly, an early modelling study [16] applied to vaccination the concept of interactions between repeat immunising exposures, illustrating that strain-matched vaccination against drifted influenza strains could be compromised in its effectiveness, by cross-reactivity with earlier vaccination. These proposed dynamics are supported by recent, multi-annual studies of the effectiveness of routine influenza vaccination [17].

Meanwhile, human cohort studies have provided valuable opportunities for resolving some of the details of longitudinal antibody dynamics. The FluScape study in Guangzhou, China [18], offered evidence for an antibody response that is hierarchical with respect to the sequence of exposures undergone by an individual, preferentially boosting immune memory to strains experienced earlier in life. This pattern has been described as antigenic seniority. Analysis of the data using mathematical modelling has lent support to mechanisms involving such preferential boosting, together with cross-reactivity between drifted strains decaying by half over only 2-3 years [19].

Recently, evidence has also emerged of protection against severe disease in antigenically novel, zoonotic viruses [3]. In particular, while the avian H5N1 and H7N9 viruses have not circulated widely in the human population, they belong to the same phylogenetic groups as human H1 – H3 viruses. Childhood exposure to H1 and H2 viruses is associated with clinical protection against avian H5N1 later in life, and likewise for protection conferred by H3 viruses, against avian H7N9. These findings illustrate that pandemic protection could be profoundly affected by early exposure to influenza viruses already circulating in the human population, although the influence of this antigenic ‘imprinting’ may be stronger against pandemic than seasonal viruses [20].

All of these studies have focused on the long-term dynamics of humoral immunity, and specifically on anti-HA immunity. However, little is known about the long-term dynamics of the cellular immune response. For example, what is the duration of this response in the absence of boosting? Do the phenomena of original antigenic seniority, or antigenic imprinting, extend to cell-mediated immunity? A challenge in addressing these questions is the difficulty of assaying cellular immunity to the same extent as humoral immunity [21]. Overall, these represent key questions of relevance to future vaccine development.

The complexity of these mechanisms mean that it is difficult to incorporate herd immunity against pandemic influenza easily into models of past exposures. In our present work we have taken a necessarily oversimplified approach ignoring all of these potential mechanisms (see above comments on ‘all-or-nothing’ immunity), in order to present a model serving an illustrative purpose. Future modelling analysis will benefit from more robust population models, taking into account the various mechanisms described above.

1. **Supplementary results**

Figure 1 in the main text illustrates how a UIV programme could increase a population’s vulnerability to a pandemic. While the figure shows results aggregated over the population, figure S2 below shows these dynamics disaggregated by age, illustrating that each of the age groups likewise experience increased vulnerability to a pandemic, under conditions of low UIV coverage.


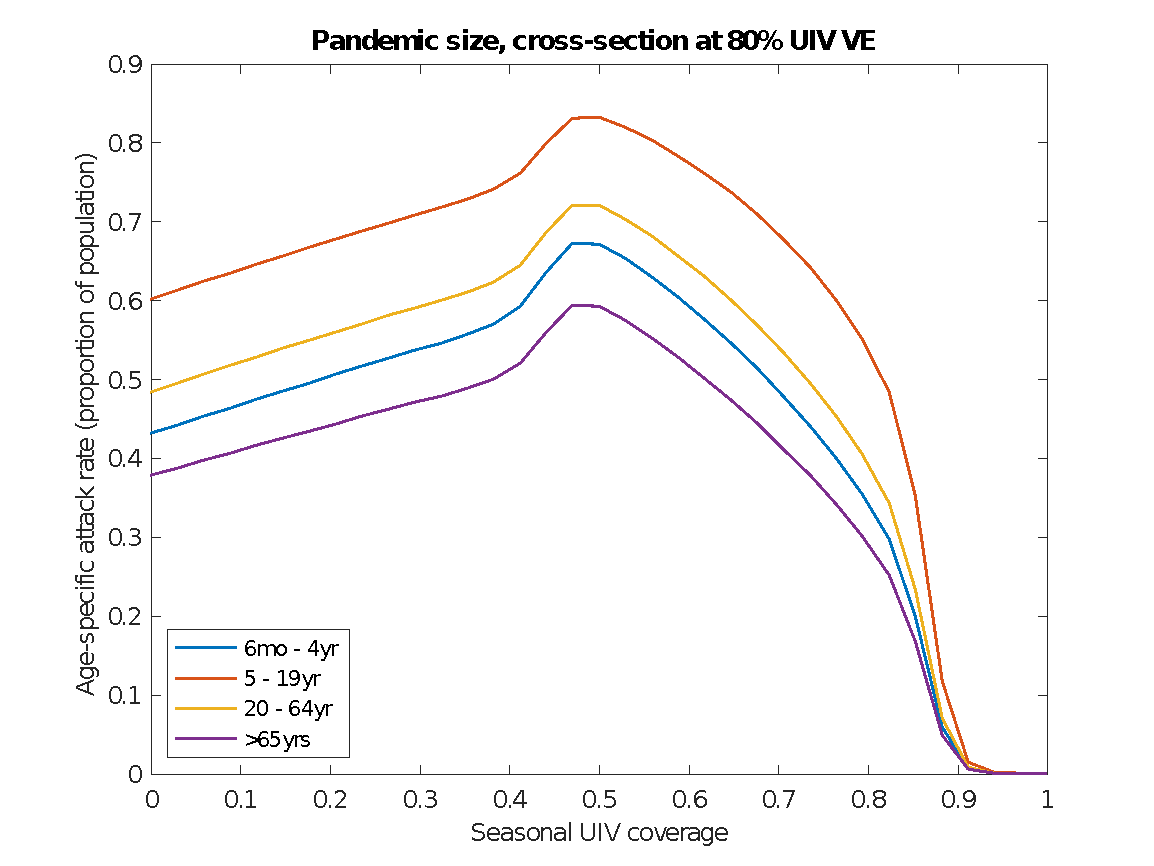


**Figure S2. Disaggregation of Figure 1 (main text) by age**. Shown is the pandemic attack rate under a scenario of 80% UIV (corresponding to the edge marked (i) in Figure 1). Different curves represent the age-specific attack rates in the different age groups, as a function of seasonal UIV coverage (horizontal axis).

**References**

1. Arinaminpathy N, Kim IK, Gargiullo P, Haber M, Foppa IM, Gambhir M, Bresee J. 2017 Estimating Direct and Indirect Protective Effect of Influenza Vaccination in the United States. *Am. J. Epidemiol.* **186**. (doi:10.1093/aje/kwx037)

2. Halloran ME, Haber M, Longini IM. 1992 Interpretation and estimation of vaccine efficacy under heterogeneity. *Am. J. Epidemiol.* (doi:10.1093/oxfordjournals.aje.a116498)

3. Gostic KM, Ambrose M, Worobey M, Lloyd-Smith JO. 2016 Potent protection against H5N1 and H7N9 influenza via childhood hemagglutinin imprinting. *Science* **354**, 722–726. (doi:10.1126/science.aag1322)

4. Russell CA *et al.* 2008 The Global Circulation of Seasonal Influenza A (H3N2) Viruses. *Science (80-. ).* **320**, 340–346. (doi:10.1126/science.1154137)

5. Lemey P *et al.* 2014 Unifying viral genetics and human transportation data to predict the global transmission dynamics of human influenza H3N2. *PLoS Pathog.* **10**, e1003932. (doi:10.1371/journal.ppat.1003932)

6. Mossong J *et al.* 2008 Social Contacts and Mixing Patterns Relevant to the Spread of Infectious Diseases. *PLoS Med.* **5**, e74. (doi:10.1371/journal.pmed.0050074)

7. Alam S, Knowlden ZAG, Sangster MY, Sant AJ. 2014 CD4 T cell help is limiting and selective during the primary B cell response to influenza virus infection. *J. Virol.* **88**, 314–24. (doi:10.1128/JVI.02077-13)

8. León B, Bradley JE, Lund FE, Randall TD, Ballesteros-Tato A. 2014 FoxP3+ regulatory T cells promote influenza-specific Tfh responses by controlling IL-2 availability. *Nat. Commun.* **5**, 3495. (doi:10.1038/ncomms4495)

9. Subramanian R, Graham AL, Grenfell BT, Arinaminpathy N. 2016 Universal or Specific? A Modeling-Based Comparison of Broad-Spectrum Influenza Vaccines against Conventional, Strain-Matched Vaccines. *PLoS Comput. Biol.* **12**. (doi:10.1371/journal.pcbi.1005204)

10. Ferguson NM, Galvani AP, Bush RM. 2003 Ecological and immunological determinants of influenza evolution. *Nature* **422**, 428–433. (doi:10.1038/nature01509)

11. Angeletti D, Yewdell JW. 2018 Is It Possible to Develop a “Universal” Influenza Virus Vaccine? *Cold Spring Harb. Perspect. Biol.* **10**, a028852. (doi:10.1101/cshperspect.a028852)

12. Arinaminpathy N, Ratmann O, Koelle K, Epstein SL, Price GE, Viboud C, Miller MA, Grenfell BT. 2012 Impact of cross-protective vaccines on epidemiological and evolutionary dynamics of influenza. *Proc. Natl. Acad. Sci. U. S. A.* **109**. (doi:10.1073/pnas.1113342109)

13. DAVENPORT FM, HENNESSY A V., FRANCIS T. 1953 Epidemiologic and immunologic significance of age distribution of antibody to antigenic variants of influenza virus. *J. Exp. Med.* (doi:10.1084/jem.98.6.641)

14. Henry C, Palm AKE, Krammer F, Wilson PC. 2018 From Original Antigenic Sin to the Universal Influenza Virus Vaccine. *Trends Immunol.* (doi:10.1016/j.it.2017.08.003)

15. Francis TJ. 1960 On the Doctrine of Original Antigenic Sin. *Proc. Am. Philos. Soc.* (doi:10.1016/S0016-0032(38)92229-X)

16. Smith DJ, Forrest S, Ackley DH, Perelson AS. 1999 Variable efficacy of repeated annual influenza vaccination. *Proc. Natl. Acad. Sci. U. S. A.* (doi:10.1073/pnas.96.24.14001)

17. McLean HQ, Thompson MG, Sundaram ME, Meece JK, McClure DL, Friedrich TC, Belongia EA. 2014 Impact of repeated vaccination on vaccine effectiveness against influenza A(H3N2) and B during 8 seasons. *Clin. Infect. Dis.* (doi:10.1093/cid/ciu680)

18. Lessler J, Riley S, Read JM, Wang S, Zhu H, Smith GJD, Guan Y, Jiang CQ, Cummings DAT. 2012 Evidence for Antigenic Seniority in Influenza A (H3N2) Antibody Responses in Southern China. *PLoS Pathog.* **8**, e1002802. (doi:10.1371/journal.ppat.1002802)

19. Kucharski AJ, Lessler J, Read JM, Zhu H, Jiang CQ, Guan Y, Cummings DAT, Riley S. 2015 Estimating the Life Course of Influenza A(H3N2) Antibody Responses from Cross-Sectional Data. *PLoS Biol.* (doi:10.1371/journal.pbio.1002082)

20. Ranjeva S, Subramanian R, Fang VJ, Leung GM, Ip DKM, Perera RAPM, Peiris JSM, Cowling BJ, Cobey S. 2019 Age-specific differences in the dynamics of protective immunity to influenza. *Nat. Commun.* (doi:10.1038/s41467-019-09652-6)

21. Coughlan L, Lambe T. 2015 Measuring cellular immunity to influenza: Methods of detection, applications and challenges. *Vaccines*. (doi:10.3390/vaccines3020293)
